# Treatment Options for Patients with Non-Small Cell Lung Cancer and Liver Metastases

**DOI:** 10.3390/cimb46120802

**Published:** 2024-11-24

**Authors:** Vesna Ćeriman Krstić, Natalija Samardžić, Milija Gajić, Milan Savić, Biljana Šeha, Marina Roksandić Milenković, Dragana Jovanović

**Affiliations:** 1 Faculty of Medicine, University of Belgrade, 11000 Belgrade, Serbia; drmilansavic@gmail.com; 2 Clinic for Pulmonology, University Clinical Center of Serbia, 11000 Belgrade, Serbia; natalis.dm@gmail.com (N.S.); milijagajic@gmail.com (M.G.); 3 Clinic for Thoracic Surgery, University Clinical Center of Serbia, 11000 Belgrade, Serbia; 4 Clinic for Neurosurgery, University Clinical Center of Serbia, 11000 Belgrade, Serbia; biljana.seha@yahoo.com; 5 Municipal Institute for Lung Diseases and TB, 11000 Belgrade, Serbia; dr.marr@gmail.com; 6 Internal Medicine Clinic „Akta Medica“, 11000 Belgrade, Serbia; draganajv@yahoo.com

**Keywords:** NSCLC, liver metastases, immunotherapy, combination therapies, radiotherapy, surgery

## Abstract

Lung cancer represents the most common cause of cancer-related death. Patients with non-small cell lung cancer (NSCLC) and liver metastases have worse prognosis, with an overall survival (OS) from three to six months. The majority of them have a poor response to chemotherapy, and the data are controversial regarding the response to immunotherapy. This could be because the liver is considered to be an immune-tolerant organ, which is characterized by T-cell anergy and immunosuppressive signals. This review evaluates current treatment options for patients with NSCLC and liver metastases. Combination therapies might be a better treatment option for this subgroup of patients. The addition of radiotherapy to immunotherapy could also be an option in selected patients. The resection of single liver metastasis should also be considered.

## 1. Introduction

Lung cancer is the most common cause of death among all cancers [1]. Non-small cell lung cancer (NSCLC) represents around 85% of all lung cancers [1]. In more than half of patients, the disease is diagnosed in the locally advanced or metastatic stage [1]. In the past, the standard of care for those patients was platinum doublet, with a 5-year survival rate of less than 10% [1].

Patients with NSCLC and liver metastases (LMs) have worse prognosis, and they make up about 20% of patients [2]. The studies have shown that these patients live approximately three to six months [2]. The results of one large study which involved more than 20,000 lung cancer patients showed that patients with liver metastases had a 53% higher risk of death compared to patients with brain metastases [3,4]. Similar results were found in the other studies, which also found that LM was associated with worse outcomes [5,6].

A large cohort study investigated which metastatic site was the most prevalent in patients with NSCLC, median time to develop metastasis and prognosis by metastatic site [7]. It was found that the most prevalent metastatic site was lungs. Regarding LM, it was found that those patients had a lower 6-month survival rate compared to patients with brain metastases [7].

Further, Akamine et al. [8] conducted a study to estimate post-recurrence rate, time to recurrence, and the impact of the site of recurrence on post-recurrence survival in patients who had radical surgery due to NSCLC. They found that liver was a significantly unfavorable prognostic site and that recurrence occurred usually within 6 months after surgery [8]. Also, it was found that LM was associated with poor post-recurrence survival; HR 2.20 [8].

In other study conducted by Takeuchi et al. [9], LM, ECOG PS ≥ 2, and non-smoking history were identified as early mortality factors in patients with advanced or metastatic NSCLC treated with mono-immunotherapy.

Deng et al. [10] found that patients with NSCLC and liver metastases had worse organ-specific response to immunotherapy compared to patients with NSCLC and pulmonary metastases. Interestingly, one third of patients had mixed responses to immunotherapy, and among them, the majority had a response in the lungs, but progression in the liver, and others had the opposite response [10]. Further, 27 paired tumor samples from pulmonary metastases and liver metastases were collected to evaluate organ specific differences. There were no significant difference in the consistency of genetic variation, tumor mutation burden, and different gene mutations [10], but lower levels of immune activation and infiltration were found in the tumor microenvironment of the liver metastases [10].

The majority of patients with liver metastases have a poor response to chemotherapy [2], but the response to immunotherapy is controversial. It has also been shown that the groups of patients with driver mutations have a worse response to targeted therapies compared to patients without liver metastases [4,11,12]. Also, the results of some studies suggested that patients with NSCLC and liver metastases respond to the treatment more similarly to patients with primary liver cancer [10,13].

Since it has been shown that patients with NSCLC and LM have worse prognosis compared to patients with other metastatic sites, even compared to patients with brain metastases, we are going to summarize the possible treatment options for this group of patients. However, the underlying mechanisms as to why these patients have a worse treatment response are sill unclear.

## 2. The Liver as an Immune-Tolerant Organ

The liver is considered as an immune-tolerant organ, which is characterized by T-cell anergy and immunosuppressive signals [10]. This could be the reason why patients with NSCLC and liver metastases have a worse response to PD-1/PD-L1 inhibitors compared to patients without [2]. Studies also have shown that liver metastases could actively induce the apoptosis of CD8+ T-cells after their interaction with macrophages, which means that liver metastases could take over the mechanisms of peripheral tolerance, leading to acquired resistance to immunotherapy through the removal of CD8+ T-cells [10].

PD-L1 is an immune checkpoint that limits T-cell activity by binding to its receptor PD-1 on activated T-cells [10]. PD-L1 can also be found on tumor cells and, as a result, blocks the ability of the immune system to fight against various cancers [10]. Furthermore, blocking PD-1/PD-L1 leads to the reactivation of exhausted T-cells and to the reactivation of the immune system, enabling it to fight against tumors [10]. In this specific process, tumors and the organ-specific microenvironment play important roles [10]. As was already mentioned, the liver is considered to be an immune-tolerant organ, and it is likely that liver metastases will be resistant to immunotherapy [10].

Vascular endothelial growth factor (VEGF) and its receptors have a crucial role in normal and pathologic angiogenesis. By activating the VEGF/VEGFR axis, other signaling pathways are triggered, which further results in endothelial cell survival, mitogenesis, migration, and differentiation, as well as vascular permeability and the mobilization of endothelial progenitor cells from the bone marrow into the peripheral circulation [14]. Further VEGF leads to increased vascular permeability and thus to the development of malignant effusions. Increased permeability can also lead to the deposition of proteins in the interstitium and, in that way, facilitate angiogenesis [14]. Finally, the overexpression of VEGF has been associated with disease progression and worse prognosis in several tumors, including lung cancer [14]. Thus, VEGF could block antitumor activity by promoting vascularization [14], which is often used by tumors for growth and metastasis and interrupting T-cell infiltration into the tumor [15,16].

It has been suggested that a VEGF inhibitor in combination with chemotherapy could enhance PD-L1 inhibitor-induced T-cell-mediated cancer cell death, by reversing VEGF-induced immunosuppression by promoting the infiltration of T-cells into the tumor microenvironment and enabling T-cell activity against tumor antigens, alongside chemotherapy-induced cancer cell death [13].

## 3. The Impact of Co-Mutations

Recent studies had shown that the presence of co-mutations also had an impact on immunotherapy efficacy. Sclera et al. [17] investigated whether KEAP1 and TP53 (a tumor suppressor gene) have an impact on immunotherapy efficacy. The KEAP1/NFE2L2 pathway has a role in the protection of eukaryotic cells from oxidative stress and xenobiotics [17]. When cells are exposed to stress, KEAP1 enables NFE2L2 to translocate to the nucleus. Further, NFE2L2 initiates a transcriptional program that leads to a cytoprotective response through the modulation of genes encoding antioxidant proteins, detoxifying enzymes, and multidrug resistance proteins [17]. Cancer cells attack this process, in order to prohibit anticancer treatment [17]. Thus, it is speculated that inadequate KEAP1 function leads to uncontrolled NFE2L2 transcription and enhanced cytoprotection [17]. Accordingly, lung adenocarcinoma-carrying KEAP1 mutations are resistant to chemotherapy and radiotherapy [17]. However, regarding immunotherapy, the data are inconsistent. TP53, along with another tumor suppressor gene, CHEK2, have a role in testing whether DNA mutations in damaged cells can be repaired or whether the cell has to be eliminated [18]. Thus, it was concluded that the presence of other co-mutations may contribute to the immunotherapy response [17,18]. KRAS mutations (among the most common mutations in lung cancer), for example, induce the hyper-activation of downstream signaling, controlling cell proliferation [18]. STK11 has a major role in lung cancer differentiation and metastasis [18].

Scalera et al. [17] found that the presence of KEAP1 and TP53 had different impacts on immunotherapy efficacy. Their results suggested that tumors with KEAP1 mutations had the shortest survival, while tumors with a KEAP1/TP53 or only a TP53 mutation had intermediate survival, and the best prognosis was achieved by tumors without any of these two mutations [17]. Interestingly, it was observed that tumors with KEAP1 and tumors with KEAP1/TP53 were entirely different in terms of mutational repertoire, the degree of intra-tumor heterogeneity, evolutionary trajectories, pathway-level signatures, and immune microenvironment [17].

Further, Wang et al. [18] found that the presence of co-mutations might have an impact on immunotherapy efficacy in patients with NSCLC. The majority of patients were treated with mono-immunotherapy [18]. Thus, they found that patients with a TP53 mutation had significantly longer PFS compared to the TP53 wild-type, and a similar observation was found in a combination group, but the difference was not significant [18]. Also, a longer PFS was found if other co-mutations were found with TP53 [18]. Thus, it was suggested that TP53 could be the dominant mutation, which correlated with longer PFS in patients with NSCLC treated with mono-immunotherapy [18]. It was also suggested that different genes could have different effects when co-mutated with TP53 [18]. Thus, it was found that patients with a co-mutation of TP53 with STK11 had longer PFS compared to the wild-type group, but without statistical significance [18].

FAT3 has a role in encoding large proteins with extracellular Cadherin repeats, EGF-like domains, and Laminin G-like domains [19]. It is involved in tumor suppression and planar cell polarity. Further FAT3 mutations have been linked to prognosis and an elevated TMB level in multiple cancers, including NSCLC [19]. LRP1B, the tumor suppressor gene, encodes the low-density lipoprotein family receptor [19]. LRP1B’s correlation with TMB and immunotherapy efficacy has been confirmed in multiple cancers, including lung cancer [19]. One study suggested that the co-mutation of FAT3 and LRP1B led to significantly prolonged PFS in patients with lung adenocarcinoma treated with immunotherapy [19], and Ying et al. [20] found that patients with squamous cell lung carcinoma and TP53 and TTN (one of the most commonly mutated genes; its role in cancer development is unclear) co-mutation had higher tumor-mutation burden levels and a better response to immunotherapy. The data regarding CDKN2A/B (tumor suppressor gene) loss have been controversial. Some results suggested that CDKN2A/B loss was associated with worse outcomes with immunotherapy [21,22], while in the study of Adib et al. [23], an association was not found.

It might also be that the presence of some other co-mutations led to the generation of LM, and thus contributed to resistance to treatment.

Thus, it was found that USP22, ID1, NT5DC2, and AK4, all together, could modulate the expression of proteins which were responsible for proliferation, angiogenesis, and the epithelial-to-mesenchymal transition in cancer cells or the hepatic microenvironment [24,25,26,27,28,29]. Thus, this modulation led to emergence of LMs and their growth [24,25,26,27,28,29].

## 4. Mono-Immunotherapy

In a pooled analysis of CheckMate 017 and 057 [30], approximately 23% of the patients had liver metastases. After a 3-year follow up, it was shown that the patients treated with nivolumab had improved OS compared to patients treated with docetaxel [30]. In the updated five-year analysis, the benefit was still observed in this group of patients; HR 0.67 [31].

Qiao et al. [32] investigated whether the site of metastases and their number were associated with the efficacy of immunotherapy in patients with NSCLC. They showed that patients with liver metastases had the shortest PFS and the shortest OS, numerically, compared to patients with other sites of metastases [32]. Also, they tested tumor specimens from 46 patients for PD-L1 expression, with the CD8+ tumor infiltrating lymphocyte density. They found that patients with liver metastases had a lower proportion of PD-L1+CD8+TIL+ tumors compared to patients without LM [32]. Patients who were treated with immunotherapy-based combination therapy had better PFS and OS (numerically) compared to patients treated with mono-immunotherapy [32].

Tumeh et al. [33] conducted research to evaluate the efficacy of pembrolizumab in patients with melanoma and NSCLC who had liver metastases. The results showed significantly reduced PFS and OS in a group of patients with liver metastases compared to those without liver metastases, in both groups of patients [33]. In the melanoma group, the optional biopsy was performed in 62 patients, and the results showed that patients with liver metastases had reduced CD8+ T-cell density at the invasive tumor margin [33]. Also, fewer CD8+T-cells were found in a group of patients who did not have a response to immunotherapy compared to patients who did have a response to the treatment [33]. Further, patients with LM had significantly lower densities of CD8+ T-cells in the other metastatic sites [33]. However, in the same samples, PD-1 and PD-L1 expression did not significantly differ between patients with and without LM [33].

Xie at al. [34] analyzed the efficacy of PD-1/PD-L1 inhibitors in patients with NSCLC and liver metastases. They found that immunotherapy was effective in patients with liver metastases, but the efficacy was inferior compared to patients without liver metastases [34]. Also, they found that patients with liver metastases had considerably lower CD8+ T-cell infiltration compared to those without LM [34].

Tian et al. [35] conducted a meta-analysis to elucidate the relationship between liver metastases and survival outcomes in patients with different tumor types who were treated with immunotherapy. The results showed worse outcomes in patients with liver metastases [35], and patients with urinary tumors had the worse outcomes, followed by patients with melanoma and NSCLC [35].

Sridhar et al. [36] presented similar findings. They analyzed the results from two trials to investigate the prognostic role of liver metastases in patients with advanced and metastatic NSCLC treated with durvalumab [36]. Liver metastases were found in 31.6% and 17.9% of patients, respectively [36]. They found, in both studies, that liver metastases were a negative independent prognostic factor, regardless of PD-L1 status [36].

Another large meta-analysis, which included 66 studies with previously treated patients with NSCLC, estimated the OS of immunotherapy in the second and further treatment lines [37]. Four studies reported OS in patients with LM, and these studies included patients treated with nivolumab [37]. OS ranged from 3.6 months to 7.5 months, and it was worse compared to patients without LM [37]. Further, a significant association was reported between the presence of LM and worse OS in three out of four studies [37]. Regarding OS rate, a 1-year OS rate was reported in three studies, and there were no data about 2- and 3-year OS rates [37]. The pooled 1-year OS rate was 23.8% [37].

Huang et al. [38] conducted a meta-analysis to evaluate the outcomes in patients with NSCLC who were treated with PD-1 inhibitors, and also to determine whether different metastatic sites had an impact on the outcomes [38]. They found that 17% of patients had liver metastases, and they had worse OS compared to those without LM [38].

The results of another study, which involved 215 patients with NSCLC who were treated with immunotherapy, showed that patients with liver metastases (19.1%) had a lower response to the therapy and also shorter PFS and OS [39].

In a retrospective analysis, Komiya et al. [40] showed that patients with liver metastases do benefit from immunotherapy, but they had inferior outcomes compared to patients without LM.

A large retrospective study included 1470 patients with NSCLC who were treated with targeted therapy, chemotherapy, and immunotherapy [41]. LMs were found in 15.9% of patients [41]. There was no difference in OS between patients with and without liver metastases who were treated with immunotherapy [41]. However, patients with liver metastases treated with immunotherapy had longer OS compared to patients who were treated with chemotherapy [41]. Patients treated with chemotherapy and targeted therapy with liver metastases had worse outcomes compared to those without LM [41].

A retrospective study investigated the efficacy of nivolumab in 201 patients with NSCLC, and among them, 29 had liver metastases [42]. The PFS and response rate were lower in patients with liver metastases [42], and it was also shown that liver metastases were associated with younger age, poorer ECOG PS, and a greater number of metastatic sites [42].

The results of the other study showed that PFS was worse in patients with liver metastases compared to those without LMs who were treated with nivolumab [43]. Similar results were presented in two other studies [44,45], and in a study by Topalian et al. [46], the five-year OS rate for NSCLC patients treated with nivolumab was lower compared to patients with other carcinomas. All patients with liver metastases had decreased survival [46].

Maugeais et al. [47] investigated whether LMs and the number of metastatic sites had an impact on immunotherapy efficacy in patients with different solid tumors. A total of 759 patients were included; among them, 71% had NSCLC, 22% had baseline LMs, and 49% had more than one metastatic site [47]. Patients with LMs had worse PFS and OS compared to patients without LMs, but the outcomes in this group of patients were not worse compared to the group of patients with other metastatic sites such as brain, bone, or lung [47]. The results were consistent in a group of patients with NSCLC [47].

A large multicenter retrospective study enrolled 291 patients with different tumors who were treated with immunotherapy [48]. More than half of patients had NSCLC [48]. It was shown that the presence of LMs and brain metastases was associated with lower PFS and OS, regardless of the histology [48].

Another retrospective study investigated the association between sites of metastatic disease and clinical outcomes in patients treated with immunotherapy [49]. A total of 90 patients were included, and only 20% of patients had lung, head, and neck cancer (there was no information about the percentage of lung cancer patients only) [49]. However, the results were similar to the previous study. Patients with LMs had worse PFS and OS compared to the patients without LMs [49].

Table 1 shows the outcomes for patients with LM treated with mono- immunotherapy in some studies. 

## 5. Immunotherapy Combinations

### 5.1. Immunotherapy + VEGF Inhibitor + Chemotherapy

An ImPower150 study [13] investigated the efficacy of different therapeutic options in patients with metastatic NSCLC in the first-line setting. It was shown that patients with liver metastases had better outcomes if they were treated with bevacizumab (a VEGF inhibitor), atezolizumab (a PD-L1 inhibitor) and chemotherapy compared to those who were treated with bevacizumab and chemotherapy alone (progression-free survival of 7.4 vs. 4.9 months; overall survival of 13.3 vs. 9.4 months) [13]. There was no difference in outcomes for patients who were treated with atezolizumab and chemotherapy compared to those treated with bevacizumab and chemotherapy [13].

It could be assumed that susceptibility to the VEGF inhibitor in patients with NSCLC and liver metastases arises from the reversal of immune suppression by the VEGF inhibitor, which basically has reduced-infiltration CD8+ T-cells, which may further enhance the T-cell-mediated killing of cancer cells by the PD-L1 inhibitor [13]. This efficacy was shown regardless of PD-L1 expression [13]. This could be explained by the activation of both mechanisms—T-cell activation by atezolizumab and interferon, and the γ-mediated induction of PD-L1 expression through the reprograming of the tumor microenvironment into the active immune system by the VEGFR inhibitor [13]. The efficacy of bevacizumab and atezolizumab in combination with chemotherapy in patients with NSCLC and liver metastases is supported by an investigation in patients with unresectable hepatocellular carcinoma who were treated with bevacizumab and atezolizumab in combination with chemotherapy [13]. It seems that patients with NSCLC and liver metastases respond to the treatment more similarly to patients with primary liver cancer [13].

### 5.2. Immunotherapy + Chemotherapy

A KEYNOTE 189 [50] study was investigating the efficacy of pembrolizumab versus a placebo in combination with pemetrexed–platinum in patients with metastatic NSCLC. The results showed that patients who were treated with pembrolizumab and pemetrexed–platinum had a significantly improved overall survival (OS), progression-free survival (PFS), and overall response rate (ORR) compared to the control group of patients, regardless of PD-L1 expression [50]. This study also evaluated the efficacy of pembrolizumab and pemetrexed–platinum in patients with liver metastases. The results showed improved OS and PFS in the subset of patients with liver metastases who were treated with the combination therapy, and the results were similar to those without liver metastases [50].

Matsumoto et al. [51] evaluated the efficacy of pembrolizumab mono-therapy compared to the combination of pembrolizumab with chemotherapy in patients with unresectable NSCLC [51]. The results of their research suggested that patients with liver metastases may benefit more from pembrolizumab monotherapy [51].

Another retrospective study evaluated the efficacy of mono-immunotherapy compared to the combination therapy—immunotherapy and chemotherapy—in 1133 patients with NSCLC [52]. Six hundred and seventy-five patients were treated in the first-line setting, 13.3% of them had liver metastases, and there were 14.2% patients with LM in the overall study population. There were no differences in PFS and OS between patients treated with mono-immunotherapy compared to patients treated with the combination therapy [52]. In contrast to the previous study, it was found that liver metastases could be a predictor for early disease progression, among others, in a group of patients treated with mono-immunotherapy, and this was not shown in a group of patients treated with the combination therapy [52]. Further, it was found that STK11 alterations were associated with lower PD-L1 expression, and TP53, TERT, and NF1 alterations were associated with high PD-L1 expression [52]. Also, it was found that STK11 loss and JAK2 alterations were potential predictors of early progression on mono-immunotherapy, and CDKN2A alterations were associated with worse long-term outcomes with the combination treatment [52].

Chen et al. [53] evaluated whether liver metastases could be a predictive indicator in patients with various advanced cancers treated with immunotherapy-based therapies. They found that 8.4% of all patients had liver metastases [53]. Patients with liver metastases had significantly shorter OS than those without LMs [53]. In a subgroup analysis, similar results were found in patients treated with mono-immunotherapy, but in patients treated with the combination therapy, OS was numerically lower, but without statistical significance [53]. In a group of patients with NSCLC, there were 18% of patients with liver metastases, and they had lower PFS and OS compared to patients without liver metastases, but the disease control rate was similar in both groups [53]. Finally, they also performed a meta-analysis to compare treatment outcomes in NSCLC patients with and without liver metastases who were treated with immunotherapy. The results showed that immunotherapy-based treatment correlated with better OS and PFS compared to the treatment with chemotherapy in both groups [53]. A subgroup analysis showed that patients with liver metastases treated with mono-immunotherapy did not have prolonged PFS compared to the patients treated with chemotherapy [53]. In contrast to those patients, patients with liver metastases treated with the combination of immunotherapy and chemotherapy did have prolonged PFS compared to patients treated with chemotherapy [53].

Another meta-analysis showed the PFS and OS benefit for patients with and without liver metastases treated with immunotherapy compared to standard therapies [54]. Also, it was shown that patients with liver metastases who were treated with immunotherapy had worse PFS compared to those without LMs, but the significance was not significant regarding OS [54].

Yin et al. [55] found in their meta-analysis that, in patients with NSCLC and liver metastases, treatment with pembrolizumab+ chemotherapy, atezolizumab+ bevacizumab+ chemotherapy, and nivolumab was superior to the other treatments regarding OS. However, regarding PFS, only atezolizumab+ bevacizumab+ chemotherapy and pembrolizumab+ chemotherapy were superior [55].

A meta-analysis conducted by Shi et al. [56] evaluated the efficacy and safety of first-line immunotherapy and chemotherapy in patients with non-squamous NSCLC. Five studies were included in the final analysis. A pooled analysis showed a significantly lower risk of disease progression and death if patients were treated with the combination of immunotherapy and chemotherapy compared to the chemotherapy alone [56]. This benefit was shown in patients with and without LM [56].

Another meta-analysis evaluated the efficacy of immunotherapy as a mono-therapy and in combination in NSCLC patients with liver, brain, or bone metastases [57]. It was found that patients with liver metastases could benefit from mono-immunotherapy as well as immunotherapy in the combination of chemotherapy plus anti-VEGF therapy, while patients with brain metastases could benefit only from immunotherapy+ chemotherapy [57].

Table 2 shows the outcomes for patients with LM treated with immunotherapy combinations in some studies. 

### 5.3. Immunotherapy + Radiotherapy

It was speculated that radiotherapy (RT) may enhance the response to immunotherapy by improving the antigen release and thus further activating CD8+ T-cells [58,59,60]. Also, it was shown that stereotactic body radiation therapy (SBRT) could activate the innate and the adaptive immune response within an irradiated tumor field and also in distant metastases [61]. This phenomenon is known as “abscopal effect”. Also, RT could increase the expression of MHC I on tumor cells and, thus, it could lead to the better recognition of antigens by cytotoxic T-cells [62].

Further, it was suggested that SBRT delivered to LM may cause a greater immune response than SBRT administrated to lung metastases [24]. In combination with CTLA-4 immune-check point inhibitors, SBRT showed clinical benefits in patients with lung or liver metastases [24].

A study evaluated the safety and efficacy of pembrolizumab with and without concurrent radiotherapy for lung and liver metastases in patients with metastatic NSCLC [63]. It was shown that patients treated with immunotherapy and RT had a better PFS, numerically, compared to patients treated with immunotherapy alone, but without statistical significance [63].

In a study which enrolled patients with oligometastatic NSCLC (including patients with liver metastases) who were treated with the locally ablative therapy of all known metastatic sites, patients were treated with pembrolizumab after 4–12 weeks [64]. The locally ablative therapy included surgery, SBRT and chemo-radiotherapy [64]. The results showed improved PFS without new adverse events [64].

In a large study which included 25 centers, 427 patients with different primary tumors and liver metastases were identified, who were then treated with SBRT [65]. Primary lung cancer was found in 12% of patients [65]. The median OS depended on the primary site of the tumor, which was 10 months for patients with lung cancer, and the one-year survival rate was 50% [65]. The median OS was also associated with the volume of metastases and the biologically effective dose [65]. Patients with liver metastases with a volume < 40 m^3^ had better OS, which was also linked with the biologically effective dose ≥ 100 Gy [65].

In a Dutch–Belgian registry, 515 patients with liver metastases were identified, who were treated with SBRT [66]. There were 8.9% patients with primary lung cancer [66]. The local control and OS rate was 87% and 84% at 1 year, 75% and 63% at 2 years, and 68% and 44% at 3 years, respectively [66]. However, the results for the local control and OS according to the primary site of the tumor were not shown [66].

## 6. Surgery

Al Farai et al. [67] detailed very interesting results. They investigated whether the resection of isolated liver metastasis had an impact on OS. The resection was performed in 23 patients, and 4 of them had NSCLC [67]. They found that the OS after the resection was 24 months, without a significant difference regarding the primary tumor site [68]. Also, they reported 1-, 3-, and 5-year survival rates of 70%, 23%, and 23%, respectively [67].

Ishige et al. [68] retrospectively evaluated the outcomes of seven patients with oligo-recurrent NSCLC in the liver, who underwent hepatic resection. The median OS after liver resection was 24 months [68].

Further, Hakoda et al. [69] reported a case of a patient who was alive 41 months after liver resection due to lung cancer metastasis, and Nagashima et al. [70] reported a case of a patient who was alive more than 5 years after liver resection due to NSCLC metastasis.

A large study was conducted in order to evaluate the outcomes in patients with metastatic NSCLC [71]. Some of the patients underwent surgery of a primary tumor or metastasis [71]. Patients with liver metastases had the worst prognosis, with a median OS of 4 months. Further, patients with only bone, brain, liver, or lung metastasis, as well as those with multiple metastases, were divided into groups depending on whether the primary tumor or metastasis was operated on [71]. So, patients with bone, brain, liver, lung metastasis, as well as those with multiple metastases, had a better outcome if they had undergone an operation of the primary tumor compared to patients who did not [71]. Similarly, this group of patients, except for patients with liver metastasis, also had a better outcome if they underwent an operation of the metastatic lesion, compared to patients who did not [71]. However, the difference was not significant in patients with liver metastasis [71].

## 7. Conclusions

As was shown, patients with liver metastases had worse prognosis compared to other groups of patients, even if they were treated with immunotherapy. As these patients represent a significant number of patients, we need different therapeutic options. It might be that combination therapies (atezolizumab+ bevacizumab+ chemotherapy; immunotherapy+ chemotherapy) represent the best therapeutic option for this subset of patients at this particular moment. Also, the addition of radiotherapy or resection could be considered for selected patients with a limited number of liver metastases.

## Figures and Tables

**Table 1:** The overview of the outcomes for patients with liver metastases treated with mono-immunotherapy in some studies.

Study	Type of Study	Applied Treatment	No of Patients; % LM	Findings
CheckMate 017 and 057-pooled analysis [31]	Clinical trial	Nivolumab vs. Docetaxel	427 vs. 427; 23.2% vs. 22%	OS HR: 0.67
Tumeh et al. [33]	Clinical trial	Pembrolizumab	165; 27.9%	LM/no LM: PFS-1.8/4.0 months
Xie et al. [34]	A real-world study	PD-1/PD-L1 inhibitor	648; 9.4%	LM/no LM: PFS-6.4/7.9 months; OS 15.2/20.6 months
Tian et al. [35]	Meta-analysis	Immunotherapy	/	OS HR: 1.81
Huang et al. [38]	Meta-analysis	PD-1 inhibitor	8067; 17%	OS HR: 1.73; PFS HR: 1.4

**Table 2:** The overview of the outcomes for patients with liver metastases treated with immunotherapy combinations in some studies.

Study	Type of Study	Applied Treatment	No of Patients; % LM	Findings
IMpower150 [13]	Clinical trial	Atezolizumab+ Bevacizumab+ Paclitaxel/Platinum vs. Atezolizumab+ Paclitaxel/Platinum vs. Bevacizumab + Paclitaxel/Platinum	1202; 10.2%	OS HR: 0.68
KN189 [50]	Clinical trial	Pembrolizumab+ Pemetrexed–Platinum vs. Pemetrexed–Platinum	616; 16.1% vs. 23.8%	OS HR (LM/no LM): 0.62/0.58
Shi et al. [56]	Meta-analysis	Immunotherapy+ chemotherapy vs. chemotherapy	/	PFS (LM/no LM): HR 0.63/HR 0.56
Yang et al. [57]	Meta-analysis	Immunotherapy combinations or mono-immunotherapy vs. chemotherapy	988; 729 pts with LM	OS HR: 0.72; PFS HR: 0.72
